# Light-Controlled Multiconfigurational Conductance Switching in a Single 1D Metal–Organic Wire

**DOI:** 10.1021/acsnano.3c12909

**Published:** 2024-03-22

**Authors:** Aleš Cahlík, Martin Ondráček, Christian Wäckerlin, Andres Pinar Solé, Olivier Siri, Martin Švec, Pavel Jelínek

**Affiliations:** † Institute of Physics of the Czech Academy of Sciences, Prague, 16200, Czech Republic; ‡ Regional Centre of Advanced Technologies and Materials, Czech Advanced Technology and Research Institute (CATRIN), Palacký University Olomouc, 78371 Olomouc, Czech Republic; § Institute of Physics, École Polytechnique Fédérale de Lausanne (EPFL), Station 3, CH-1015 Lausanne, Switzerland; ∥ Laboratory for X-ray Nanoscience and Technologies, Paul-Scherrer-Institut (PSI), CH-5232 Villigen, PSI, Switzerland; ⊥ Aix Marseille Université, CINaM UMR 7325 CNRS, Campus de Luminy, 13288 Marseille cedex 09, France; # Department of Physics, University of Zurich, Winterthurerstrasse 190, CH-8057 Zurich, Switzerland

**Keywords:** molecular chains, spin crossover, light-induced switching, scanning tunneling microscopy, transport, density functional theory, one-dimensional system

## Abstract

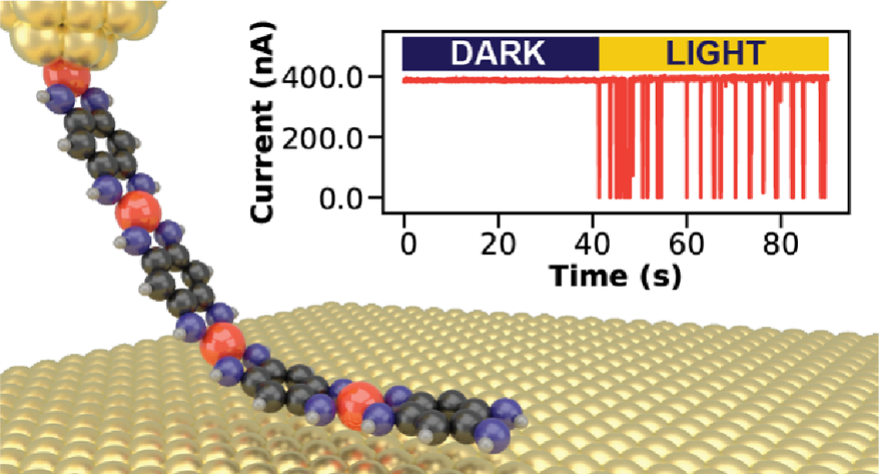

Precise control of multiple spin states on the atomic scale presents a promising avenue for designing and realizing magnetic switches. Despite substantial progress in recent decades, the challenge of achieving control over multiconfigurational reversible switches in low-dimensional nanostructures persists. Our work demonstrates multiple, fully reversible plasmon-driven spin-crossover switches in a single π-d metal–organic chain suspended between two electrodes. The plasmonic nanocavity stimulated by external visible light allows for reversible spin crossover between low- and high-spin states of different cobalt centers within the chain. We show that the distinct spin configurations remain stable for minutes under cryogenic conditions and can be nonperturbatively detected by conductance measurements. This multiconfigurational plasmon-driven spin-crossover demonstration extends the available toolset for designing optoelectrical molecular devices based on SCO compounds.

Low-dimensional quantum systems, such as metal–organic complexes, constitute an ever-growing and varied class of functional entities. Their potential stems from their prospective use in electronic or spintronic circuits and nanodevices. Primarily, this could be achieved through the precise manipulation of their magnetic and electronic properties at the atomic level. Exploring externally controlled transitions between single-molecule levels in such systems not only offers avenues for technological advancements but also allows understanding the fundamental physical and chemical mechanisms.^1,2^

One of the potential routes for realizing switching technologies relies on the spin-crossover (SCO) phenomenon,^3,4^ investigating changes in the electronic spin state of complexes induced by external stimuli.^5−7^ Light is of particular interest because it can promote three different effects depending on the role of irradiation wavelength: conformation changes (photoisomerization or photocyclization),^8^ photoinduced spin-state switching (including valence tautomerism),^9−12^ and temperature increase (photothermal effect).^13^ An interesting possibility of targeting SCO in individual molecules is the use of optical nanocavities. Recent developments in nanophotonics, in particular utilizing the strong coupling of light and matter in optical nanocavities,^14^ have enabled triggering of chemical reactions,^15^ spin,^16^ and nanomechanical^17^ switches or motion of single atoms in nanoparticles.^18^ One of the major challenges for the realization of molecular-based spintronics devices is the scalability and accessibility of spin states in a single-molecular object. However, despite large progress in nanophotonics and transport in single-molecular junctions^19^ in the past few years, the possibility of controlling multispin configuration in a single nanoobject placed in a nanocavity has not yet been demonstrated.

In our work, we show that a single cobalt-based metal–organic nanowire, suspended between two metallic electrodes, exhibits repeated light-induced conductance switches between distinct states characterized by a high on–off conductance switching ratio. By means of a scanning probe, we tune the number of these states by altering the suspended wire length. We demonstrate that the states maintain a long lifetime of several minutes once the light source is turned off, and the individual states can be read without alternation. On the basis of theoretical calculations of the electronic structure and transport, we attribute the origin of the conductance switching to multiple SCO between the low- and high-spin states of the cobalt centers. Note that so far most SCO switches are based on iron complexes.^20^ SCO systems based on Co(II) compounds are also well documented for complexes having an octahedral geometry,^21−23^ but appear much rarer in the case of square planar complexes.^24^ Namely, the control of spin states in square planar polynuclear Co(II) complexes at specific positions has hitherto been unknown.

## Results and Discussion

### Chain Manipulation and Conductivity

**Figure 1:**
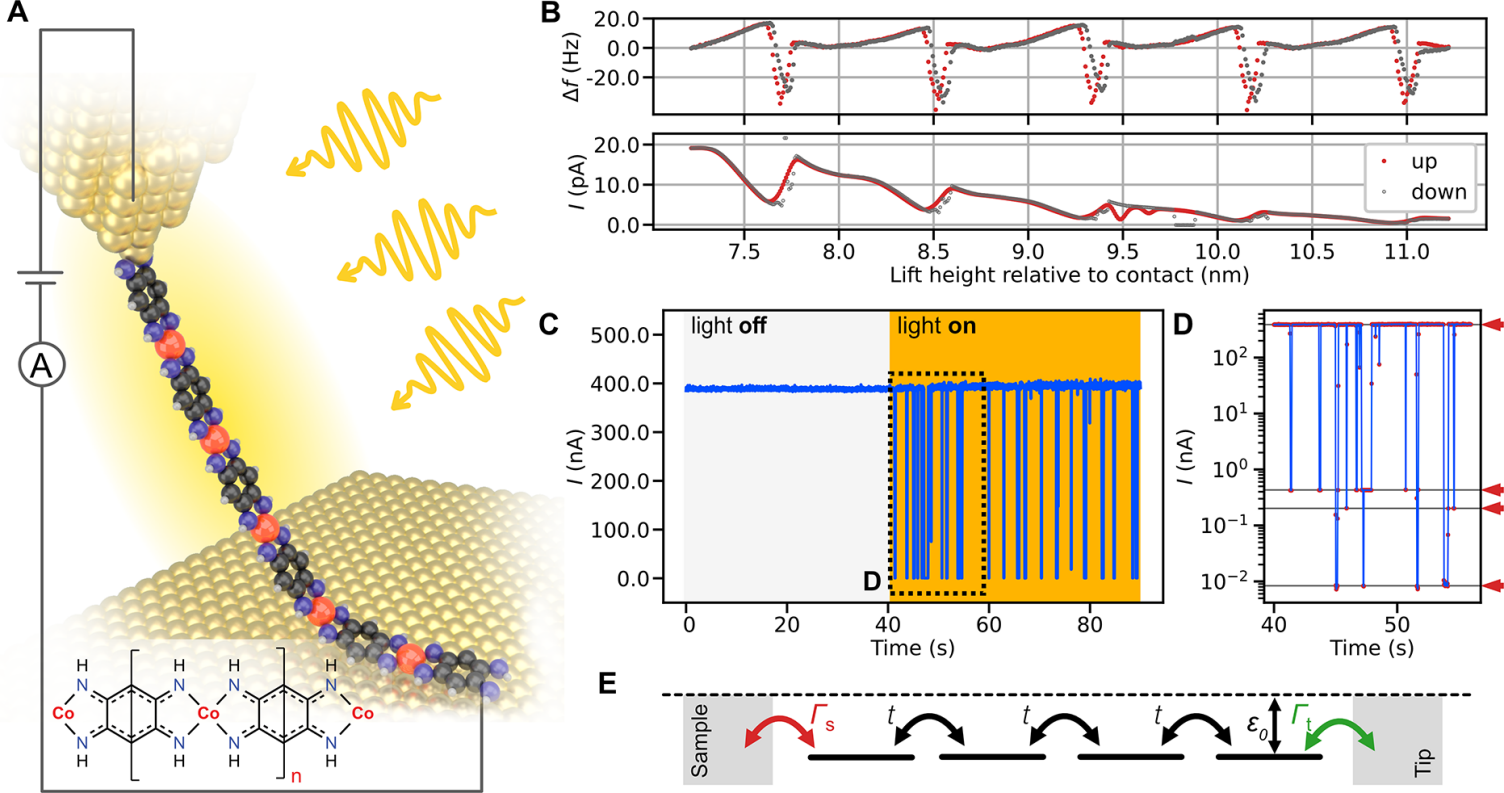
Effect of light illumination on conductance of Co-QDI chains. (A) Sketch of the experiment: a single metal–organic wire suspended between the tip of a scanning probe microscope and Au(111) sample. The combination of atomic force microscopy (AFM) and scanning tunneling microscopy (STM) allows for simultaneous observation of lifting dynamics and wire conductance via frequency shift (Δ*f*) and current (*I*) channel, respectively. Inset: Structure of the Co-QDI polymer. (B) Characteristic Δ*f* and *I* traces acquired during repeated lifting and lowering of the wire. The Δ*f* trace shows a characteristic period of ∼0.8 nm, corresponding to the Co-QDI wire periodicity (Santhini et al., 2021). The current signal recorded shows detectable current even at 11 nm lift (corresponding to ca. 13–14 units lifted, *V* = 1.3 V). (C) An example current versus time trace demonstrating the effect of junction illumination on chain conductivity (lift height ∼ 2.5 nm, *V* = 90 mV). Illuminated with monochromatic 600 nm light from a Xe lamp. (D) Zoom-in on (C) plotted in logarithmic scale revealing the presence of multiple conductance levels (indicated by red arrows). (E) Schematic sketch of the chain conductance model (detailed in the Supporting Information).

We synthesize isostructural Co-QDI chains on a Au(111) surface by co-deposition of single Co atoms and 2,5-diamino-1,4-benzoquinonediimine (QDI)^25^ precursors under ultra-high-vacuum (UHV) conditions following the procedure introduced in our previous works.^26−28^ This results in the formation of flexible and planar π–d coordination polymers consisting of 4-fold-coordinated Co atoms to QDI ligands (Figure 1A and Figure S1). The chains weakly interact with the underlying metallic Au(111) surface and can be vertically manipulated^29,30^ at cryogenic temperatures (5 K) by a scanning probe, as shown in Figure 1A (detailed in the Supporting Information). Figure 1B shows a typical frequency shift (Δ*f*) and current (*I*) signal recorded during a controlled manipulation process after gentle contact of a probe with a chain edge (1.3 V bias voltage). Both channels recorded during the process reveal a characteristic periodic signal with a period of ∼0.8 nm, which matches very well with the optimized lattice vector, *a* = 0.79 nm, of an infinite Co-QDI chain obtained from total energy density functional theory (DFT) calculations. We achieve the high congruence of up and down traces displayed in Figure 1B after a few repeated lifting and lowering cycles (in a fixed height span above the surface); nevertheless the periodic features appear already during the initial lift (Figure S2). Consequently, we used them to estimate the number of lifted molecular units in the suspended chain. During the lift, the chains exhibit a rather large conductivity. Notably, we observe a detectable current for suspended chains longer than 10 nm, and the chain’s conductance shows an unusually small decay constant λ of 1.07 nm^–1^; see Figure S2.

To explain the polymer’s high conductivity, we develop a simple theoretical transport model. The model consists of a linear chain of sites with energy *ϵ*_d_, representing selected d-orbital residing on the Co atom (as will be rationalized later), coupled by an effective hopping with the nearest neighbor sites *t*_π__–d_ or two electrodes for edge sites Γ as shown in Figure 1E (details in the Supporting Information). *t*_π–__d_ stands for an electronic coupling between the Co-localized d-orbitals through the ligand π-orbitals, forming delocalized π–d bands observed in previous ARPES measurements.^26^ With a proper choice of model parameters *ϵ*_d_ = 1.1 eV, *t*_π__–d_ = 0.5 eV, we recover the experimental value of the characteristic decay length λ = 1.08 nm^–1^. These values ensure that the top edge of the chain band is located −0.1 eV below the Fermi energy (*E*_F_ = 0). As a consequence, the proximity of the dispersive π–d valence to the Fermi level^26^ leads to the high conductivity of the suspended chain.

### Light-Induced Conductance Switching

Next, we expose the suspended Co-QDI chains to a monochromatic light source and monitor their conductivity, as illustrated in Figure 1A. When the lift height exceeds a critical value (varying between 0.7 and 3 nm for individual lift experiments), the light induces fluctuations in the current signal between high and low conducting state (Figure 1C). Surprisingly, the chain remains in the last (high or low) conductive state for tens of seconds after the light is turned off, as demonstrated in Figure S3. A closer examination of the current trace recorded during the illumination (using a logarithmic scale) reveals that the conductance switches between several distinct states (Figure 1D). By modifying the intensity of the incoming light, we note that the conductance switching rate increases linearly with the light intensity (Figure S4A.). Additionally, to study the influence of photon energy on the switching behavior, we changed the wavelength of the incoming light from 200 nm to 1100 nm. As shown in Figure S4C, we can observe a noticeable switching rate over a wide range of wavelengths from 650 to 1100 nm (the upper limit of the experimental setup). While the high-energy switching threshold around 550 nm correlates with the reflectance edge of gold,^31^ we do not observe any low-energy photon limit in the available photon energy range. Essentially, the switching rate mostly replicates all of the prominent features found in the intensity spectra of the Xe light source, with additional modulation discussed later.

**Figure 2:**
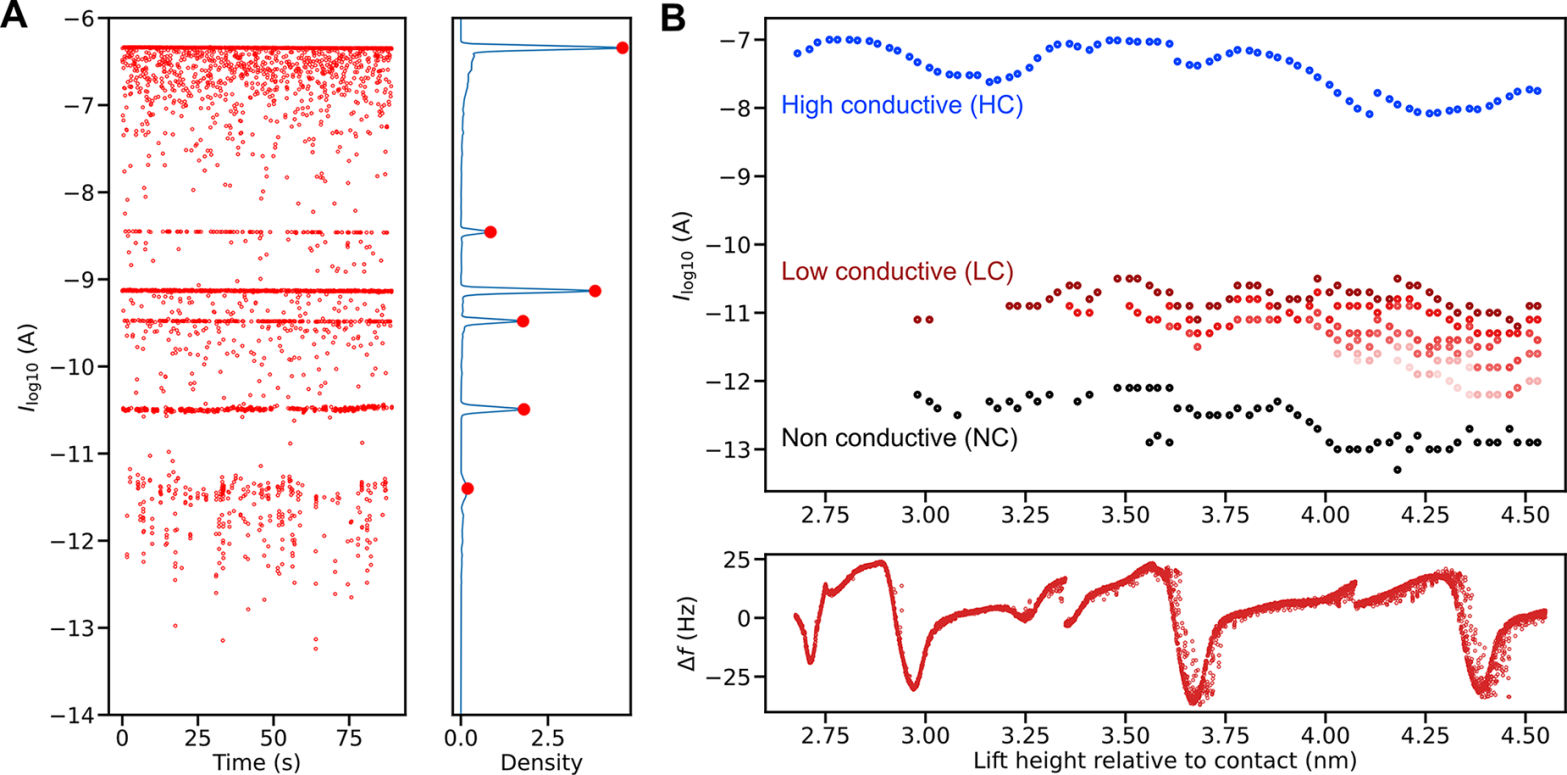
The increase in number of conductance levels with wire length. (A) Left: An exemplary current versus time trace shows switching between several conductance states. The data points localized between the conductance levels are an artifact of the 10 ms integration time (*V* = 135 mV). Right: Kernel density estimate (KDE) of the recorded trace was used for automatic identification of the accessible levels. (B) Upper: Representative experiments showing evolution of conductance levels with the lift height, extracted from the KDE for each lift increment. After reaching a critical length (3–4 units lifted), we clearly observed three different sets of conductive states: HC, LC, and NC states. While there is only one HC state independent of the length of the suspended chain, the number of LC states increases linearly with the number of units of the suspended chain. Lower: Δ*f* signal recorded during the incremental chain lifting. The trace is composed of small segments measured several minutes apart (duration of *I* vs *t* measurement, 3 min) (*V* = 5 mV during the whole experiment, Xe lamp λ = 600 nm, *t*_int_ = 20 ms).

The relatively precise control over the number of lifted molecular units enables us to systematically investigate the conductance-switching behavior as a function of the lift height (detailed in the Supporting Information). Prior to the experiment, we illuminate the junction with a monochromatic light for more than 20 min to thermally stabilize the junction. Following initial contact with the probe, we gradually lifted the polymer by small steps with a fixed bias applied (approximately 5–20 pm, varying between individual experiments) while recording the frequency shift signal. After each lifting step, we monitored the current as a function of time for several minutes to ensure a robust statistical analysis of the switching behavior. As illustrated in the representative current–time trace in Figure 2A, the polymer conductivity exhibits a distinct switching pattern between several characteristic states. By analyzing the kernel density estimate (KDE) of the recorded current–time traces (Figure 2A), we extracted the accessible current levels corresponding to each chain-length increment. This enables us to observe the evolution of accessible conductance states.

An example of such an experiment is shown in Figure 2B. To avoid potential instabilities due to high currents flowing through a short chain, the data recording starts after an initial lift of 2.6 nm from the height of the contact. Despite the light source being turned on constantly, no conductance switching is observed for short distances. Initially, we detect only one conductance state with a magnitude of 10^–7^ A, which we denote as a high-conductive (HC) state. The situation changes significantly when we lift the chain by approximately 3.0 nm (equivalent to 3–4 units), at which point two new well-defined conductive states emerge alongside the HC state, one around 10^–11^ A and another below 10^–12^ A, that we will for simplicity call low-conductive (LC) and nonconductive (NC) states, respectively. As the suspended chain is pulled further, we observe that the switching between the two (or more) distinct current states is still present. We note that while the HC state remains stable, the number of LC substates increases linearly with the number of lifted units. Although the data indicate similar behavior for the NC states, we cannot clearly differentiate between them due to the limit of detection capabilities of our experimental setup (>1 pA) and accordingly refer to them as NC. It is essential to emphasize that the critical lifting height, at which the switching starts, and the specific current magnitudes may differ among individual experiments. Nonetheless, the overall qualitative behavior remains characteristic across all the conducted experiments (examples in Figure S5).

**Figure 3:**
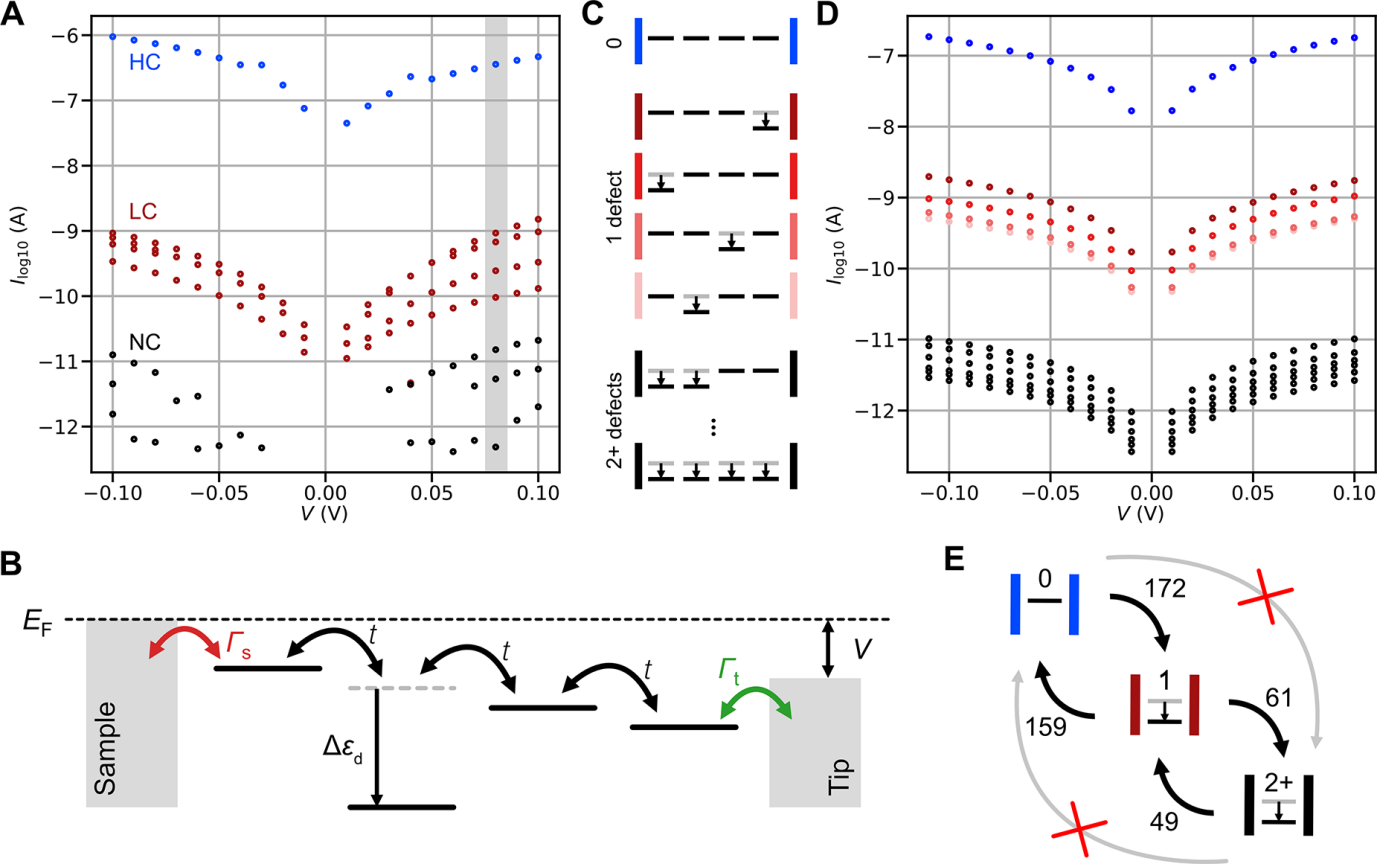
Transport model describing the conductance level structure. (A) Conductance levels in the bias range of ±100 mV observed at constant chain length (4 units) under illumination (Xe lamp λ = 600 nm, *t*_int_*=* 10 ms). Different colors were used to distinguish levels ascribed to the HC (blue), LC (red), and NC (black) states. (B) A sketch of the transport model of the suspended chain is represented by a series of Co sites connected by hopping *t*; defective sites are shifted in energy by Δε_d_. (C) Sketch of the possible defect combinations on a chain of four periodic units. (D) Simulated conductance of the chain according to the model sketched in (B) with up to two defect sites placed at various positions. These simulation results are intended to be compared with those in panel (A). (E) Statistics of the switching events that correspond to the number of defects being changed in the indicated direction, as obtained from the current vs time trace for *V* = +80 mV (marked by a gray stripe in (A), detailed in the Supporting Information).

We examined the impact of the applied bias voltage on the switching behavior of the suspended polymer. After lifting several chain units, we again record a series of current–time traces at fixed height for different bias voltages, while the light source remains on throughout the experiment. Following the same protocol as before, we perform a KDE analysis on each data set, as demonstrated in Figure 2A. Figure 3A displays a representative plot of the conductance levels versus bias voltage within the range of ±100 mV, obtained after retracting the tip by 3.0 nm, corresponding to a suspended chain of 4 units. Here, we observe three distinct conductive states—HC, LC, and NC—which evolve continuously with the applied bias voltage. In addition to the single HC state, we identify a set of four LC substates and a manifold of NC states that are partially indistinguishable due to their low-current amplitude. Notably, increasing the applied bias voltage leads to an increase in the current amplitude, enabling us to distinguish the presence of at least three NC states at 100 mV.

### DFT Calculations and Expansion of the Transport Model

**Figure 4:**
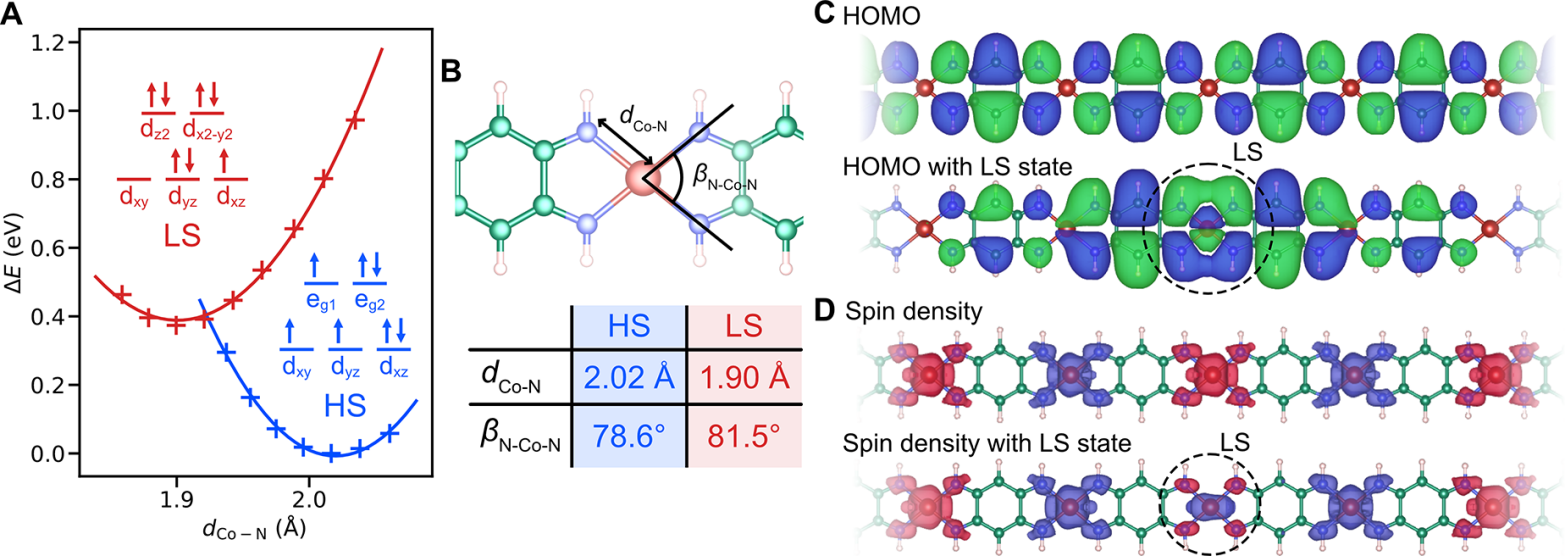
Atomic and electronic structure of the Co-QDI chain. (A) Crossover between the high-spin (HS) and low-spin (LS) state according to DFT calculations: Total energy of the respective states as a function of bond length between the central Co atom and the ligand N atoms. Inset: Spin configurations of the Co d-shell corresponding to the LS (red) and HS (blue) state, respectively. (B) Sketch of the squared planar configuration of the Co atom in the chain; comparison of the bond length (*d*_Co–N_) and angle (β_N–Co–N_) of the optimal geometries for the HS and LS state. (C) Highest-occupied molecular orbital (HOMO) for a homogeneous HS chain configuration (top) and for another HS chain configuration with one LS defect (bottom), showing strong localization of the electronic state. (D) DFT spin density for the homogeneous HS chain (top) and for the LS defect (bottom).

We rationalize the conductance switching mechanism by the well-known light-driven spin-crossover (LDSCO) mechanism (alternative scenarios discussed in the Supporting Information), which has been extensively investigated mainly across various Fe^2+^ complexes.^32^ To substantiate this proposition, we perform a theoretical analysis based on the total energy DFT calculations^33^ of a free-standing Co-QDI chain. Following the conclusion of the previous work,^28^ we identify the magnetic ground state of Co-QDI as high-spin (HS) *S* = 3/2 with in-plane magnetic anisotropy of 30 meV. Interestingly, according to hybrid DFT calculations, the low-spin (LS) doublet *S* = 1/2 magnetic state can be stabilized, and it has a slightly higher energy of only ∼0.4 eV (see Figure 4A).

The LS and HS spin states are characterized by a slightly different coordination of the Co center with the four neighboring nitrogen atoms (Figure 4A,B). Noteworthy, we find that the spin ground state energy and the energy difference between HS and LS states strongly depend on the employed portion of exact exchange–correlation in the hybrid exchange–correlation PBE0 functional^34^ (detailed in the Supporting Information, Table S2). This finding indicates the feasibility of the SCO in Co-QDI chains.

Consequently, we carry out the total energy DFT simulations of a periodic free-standing Co-QDI chain composed of 8 units where we initiate one Co site with local spin *S* = 1/2 instead of *S* = 3/2. The calculation indicates that the spin configuration remains stable after full optimization of the Co-QDI chain structure. The spin density map of the Co-QDI chain with one LS state shown in Figure 4D reveals the presence of a well-localized LS state on the specific Co atom. We also note that the configuration with two LS doublet Co-sites remains stable independently of the sites’ mutual position (Figure S6), as their interaction has a short-range character on the order of meV. These findings suggest that it is indeed feasible to stabilize a single Co site in the LS state within the Co-QDI chain.

As illustrated in Figure 4B, the LS state of individual Co sites is identifiable by a slightly different rearrangement of atomic coordinates. Notably, the Co–N bond length undergoes a reduction from 2.02 Å in the HS state to 1.9 Å in the LS state, and the N–Co–N angle changes from 78.6° (HS) to 81.5° (LS). This alteration induces a downward shift of the energy levels of the respective d-orbitals and leads to a change in their overall occupancy. The modified ligand field further stabilizes the LS state of the Co site, but most importantly, the local LS state strongly disturbs the delocalization of the HOMO orbital in both spin channels, as seen in Figure 4C, conceivably influencing the conductivity of the suspended chain, consistent with the experimental observations. The rearrangement of the localized d-orbitals on a Co atom associated with the HS to LS transition is illustrated in Figure S7.

We continue by expanding the transport model discussed earlier by incorporating the possibility of localized LS states. In this framework, we assume that the formation of an LS state can effectively lead to a reduction in the on-site energy *ϵ*_d_ at a specific site by an energy Δϵ_d_, as shown schematically in Figure 3B. To account for the presence of multiple LS states, we apply the energy shift Δ*ϵ*_d_ across a corresponding number of on-site energies *ϵ*_d_. The influence of an applied bias is illustrated through the linear shift of both the Co sites and electrodes. Using Green’s function formalism, the extended model enables the computation of the current *I* vs bias *V* curves (detailed in the Supporting Information and Figure S8). Figure 3C presents computed *I*/*V* curves for a chain model comprising 4 units, which replicate well the primary experimental findings. Namely, we can discern the three characteristic sets of the HC, LC, and NC states. A single HC branch corresponds to the case in which the model chain lacks any perturbed site. We also identify four LC branches evolving asymmetrically with applied bias. Each branch corresponds to a case in which one of the four Co sites is perturbed. Lastly, a set of NC states corresponds to the cases where two perturbed Co sites within the suspended chain are presented. A more detailed representation of results obtained for the model can be found in Figure S9.

### Switching Rates

We finally estimate the switching rates between the HC (0 → zero LS state), LC (1 → one LS state), and NC (2+ → more than two LS states) states for the suspended Co-QDI chain made of 4 units in the bias range from −100 to +100 mV from the data set presented in Figure 3A. Figure 3E exemplifies the number of switches obtained for a bias voltage of 80 meV in a time interval of 120 s. According to our analysis shown in Figure S10, we observe only sequential transitions (0 ↔ 1 and 1 ↔ 2) corresponding to the formation or destruction of a single LS state within the chain (detailed in the Supporting Information).

Figure S11 reveals no systematic dependence of switching rates of allowed transition on the applied bias. Upon analyzing the data set, we observe that the most frequent transitions happen between HC(0) and LC(1) states. In contrast, the transitions between LC(1) and NC(2+) are almost 3 times lower. The lifetime of the HC state is determined by the 0 → 1 transition (as the reciprocal of the corresponding transition rate, see Figure S11), so it is about 0.5 s. The lifetime of the LC state is dictated by the 1 → 0 rate, which falls between 20 and 30 s^–1^, resulting in an approximate lifetime of 0.04 s. We note that the rate of 2 → 1 is substantially lower than that of 1 → 0, so the probability of defect destruction (switch of the spin-defective site from the LS back to the HS configuration) is higher for a single isolated low-spin defect than for two LS defects present simultaneously. This suggests that the interaction between two LS defects appears to stabilize them.

### Mechanism of the Transition

The precise mechanism underlying the spin-crossover transition remains an open question. We assign the switching mechanism to the effect of a plasmonic nanocavity formed in the SPM junction between the metal tip and the substrate linked with the Co-QDI polymer.^35^ The presence of a plasmonic nanocavity can induce a transition between the two coordination configurations of the Co atom corresponding to the LS and HS states, either by a direct excitation, exciton–phonon coupling mechanisms, or hot carrier generation.^36−38^ It has been theoretically demonstrated that the strong coupling between molecular dipole and confined electromagnetic field can modify the ground-state energy landscape driving SCO switches.^39^ Such nanocavity-mediated switching can, in principle, occur over a wide range of wavelengths and will depend on the spectral characteristics and geometry of the nanocavity.

This plasmon-mediated SCO scenario is supported by the following observations. Alongside the sharp drop in the switching rate below 600 nm, associated with the absorption edge of gold, the switching frequency between 600 and 700 nm is increased significantly while the intensity of the incident light remains nearly constant in the entire 450–800 nm range (Figure S4C). This is consistent with the typical plasmonic response of gold nanoparticle-surface nanocavities of comparable dimensions.^40^ In addition, the switching is about 3× more efficient for p-polarized light than for s-polarized light; see Figure S4B, reflecting the inherently prevailing absorption of p-polarized light in nanocavities formed between a sharp tip and a surface.^41^

## Conclusion

This study presents the successful implementation of spin-crossover multiconfigurational switching in suspended 1D metal–organic chains by using plasmonic nanocavities. Notably, both high- and low-spin states demonstrate long lifetimes without external light for several minutes under cryogenic conditions. This work demonstrates the potential for designing optoelectronic detectors on the molecular scale using suspended chains in plasmonic nanocavities. It also expands the possibilities of plasmon chemistry to tune the chemical properties of individual molecules. We anticipate that this work will inspire further investigation of multiconfigurational spin-crossover systems in organometallic complexes driven by plasmon nanocavities.

## Methods

### Sample Preparation

The Au(111) crystal was purchased from MaTeck Material Technologie & Kristalle GmbH. The surface was prepared in a standard way by repeated Ar^+^ etching and annealing cycles. The synthesis of the precursor molecule 2,5-diamino-1,4-benzoquinonediimine (2HQDI) followed the methodology outlined in the existing literature. We dose the molecules by sublimation from a tantalum pocket resistively heated to 115 °C. To achieve the chain formation, the precursor molecule is co-deposited with cobalt atoms (EFM 3, Focus GmbH) onto the Au(111) surface heated to 300 °C.

### Experimental Setup

All experiments are performed in an ultra-high-vacuum nc-AFM/STM microscope (CreaTec Fischer and Co. GmbH) operating at temperatures of 4–7 K. The tungsten tips are mounted on a commercially purchased qPlus sensor and were shaped by gentle interaction with the Au(111) substrate. The tunneling current was measured with a variable-gain low-noise current amplifier (DLPCA-200, Femto). To perform the light-induced experiments, we use the equipment by QuantumDesign GmbH, namely, a LOT MSH-150 monochromator and an LSB523 150 W Xe ozone-free lamp. The light on the exit of the monochromator is coupled into an optical fiber (M74L02, Thorlabs GmbH) and afterward is collimated (LA4148, Thorlabs GmbH) and guided through the UHV chamber window (Sapphire UV-grade, Vacom GmbH) and small openings in the radiation shields onto the sample. The estimated spot size of the light on the Au(111) sample is 2–3 mm in diameter.

### Chain Contacting and Lifting

For most of the experiments, we use chains with a length greater than 15 nm. The SPM probe is positioned above the Au(111) substrate, several nm from a chain end (Figure S1). Afterward, the tip is manually approached without feedback toward the gold surface using a 1 mV bias voltage until the current reaches 1 nA. Consecutively, we laterally approach the chain end at constant height (the current increases to 10 nA). To ensure that the chain is well attached to the tip and to avoid strain effects, the chain is first laterally manipulated at constant height for a few nm. Then, the chain is lifted automatically (with a Z spectroscopy module) to the desired height while recording the frequency shift and current channels to gain insight into the lifting process. A successful lifting attempt creates a characteristic Δ*f* signal trace (Figure S2). To achieve concurrent up and down Δ*f* traces (Figure 1B), the chain usually needs to be lifted and lowered several times in a fixed height interval above the surface. To lay down a longer section of a chain, it is advisable to move the tip laterally in the anticipated chain direction to avoid chain folding and strain effects. A 2 V pulse is used to detach the chain completely from the probe.

### DFT Calculations

We have employed spin-polarized DFT calculations in order to show the stability of the proposed atomic structures and magnetic states of the Co-QDI chains, reveal their electronic structure, and, further, rationalize the relation between the magnetic state and the bond geometry. The data obtained from DFT are shown in Figure 4, Figure S6, and Figure S7. All DFT calculations were carried out with the FHI-AIMS code.^33^ As the exchange–correlation functional, we took the PBE0 hybrid functional,^34^ modified by changing the portion of the Fock exchange contributing to this functional (from the standard 25% to 40%). The implementation of hybrid functionals in FHI-AIMS relies on the resolution of the identity approach.^42^ We used the “light” default atomic basis set provided with the code. The system was represented by an infinite molecular chain in free space without considering the surface. The periodic supercell contained 8 Co-QDI units. The reciprocal space was sampled by 3 *k*-points in the direction along the infinite chain. The length of the supercell along the molecular chain was allowed to relax, thus effectively optimizing the length of the Co-QDI unit. The size of the supercell perpendicular to the molecular chain was set to 2 × 2 nm, enough to accommodate the width of the molecular structure and provide sufficient space to prevent spurious interaction between periodic images of the structure. All atoms in the molecular structure were allowed to relax into optimal positions, except that for the purpose of generating the energy dependence on the Co–N bond length in Figure 4A, we had constrained the projection of the Co–N distances onto the long axis of the chain. The geometry was assumed to be optimized when forces on all atoms fell under 5 meV/Å.

## Data Availability

The data underlying this study are openly available in Zenodo repository at https://doi.org/10.5281/zenodo.10853666.

## Supporting Information Available

Lift and light experiments, counting the switching events and evaluation of the switching rate, absence of the direct conductance switching between HC and NC states, conductance model, interaction between low-spin defects, exchange-functional dependence of magnetic states, alternative switching scenarios, Figures S1 to S13 (PDF)
