# Supplementary material for: Light-Controlled Multiconfigurational Conductance Switching in a Single 1D Metal–Organic Wire
Source: ACS Nano. 2024 Mar 22;18(13):9576–83. doi: 10.1021/acsnano.3c12909 (PMC10993641; doi:10.1021/acsnano.3c12909)
Supplement: Supplementary file 1 — nn3c12909_si_001.pdf [file nn3c12909_si_001.pdf]

Supporting Information for : Light-controlled multiconfigurational conductance switching in a single 1D metal-organic wire

Aleš Cahlík[1,6], Martin Ondráček[1], Christian Wäckerlin[1,3,4], Andres Pinar Solé[1], Olivier Siri[5], Martin Švec[1], and Pavel Jelínek[1,2]

[1]Institute of Physics of the Czech Academy of Sciences, Prague, 16200, Czech Republic.
[2]Regional Centre of Advanced Technologies and Materials, Czech Advanced Technology and Research Institute (CATRIN), Palacký University Olomouc, 78371 Olomouc, Czech Republic.
[3]Institute of Physics, École polytechnique fédérale de Lausanne (EPFL), Station 3, CH-1015 Lausanne, Switzerland.
[4]Laboratory for X-ray Nanoscience and Technologies, Paul-Scherrer-Institut (PSI), CH-5232 Villigen PSI, Switzerland.
[5]Aix Marseille Université, CINaM UMR 7325 CNRS, Campus de Luminy 13288 Marseille cedex 09, France.
[6]Department of Physics, University of Zurich, Winterthurerstrasse 190, CH-8057 Zurich, Switzerland.

Contents

1 Supporting text **3**

1.1 Lift & light experiments 3

1.2 Counting the switching events and evaluation of the switching rates 3

1.3 Absence of the direct conductance switching between HC and NC states . . 4

1.4 Conductance Model 5

1.5 Interaction between low-spin defects 7

1.6 Exchange-functional dependence of magnetic states 7

1.7 Alternative switching scenarios 8

2 Supporting Figures **9**

1 Supporting text

1.1 Lift & light experiments

To stabilize the thermal drift, we always illuminate the SPM junction with the light source 30 minutes prior to the experiment. If not specified otherwise, the junction is illuminated during the whole experiment with a constant light intensity. This intensity level is selected to ensure a sufficiently high switching rate, thereby generating adequate statistical data.

From the relatively smooth Δf traces presented in Fig. 2B and Fig. S5a,f, which are composed of short lifting intervals separated in time (minutes), can be inferred that the drift in the z (and probably also in x and y) direction can be considered negligible with respects of number of lifted molecular units. Furthermore, as can be seen in the exemplary current vs time traces presented in Fig. 2A and Fig. S5b-e,g-j, the magnitudes of the respective current levels are constant during the 3 min interval, indicating minor junction drift.

The bias voltage during the lift & light experiments is set to a sufficiently small value to safely fit the current signal in the range of the operational amplifier ($< 10\,\mathrm{nA}$). The integration time (denoted as t_{int} in the text) for recording the current vs time is set usually in the range of $10 - 50\,\mathrm{ms}$ as a compromise between the time resolution and size of data files. This leads to occurrence of datapoints in between the current levels in the I vs t traces (Fig. 2A and Fig. S5b-e,g-j). We utilize the scikit-learn and SciPy Python packages for Kernel Density Estimation (KDE) and detection of current levels. For all data presented in the manuscript, we employ a Gaussian kernel, with the kernel bandwidth manually adjusted for each experimental dataset.

It is important to note that during the chain lift, the chain undergoes spontaneous relaxations and changes (movement of the chain on the substrate or on the tip, chain internal relaxation) that can lead to glitches and discontinuities in the presented data sets, however, the presented observations in switching trends are reproduced repeatedly for different samples and SPM tips.

1.2 Counting the switching events and evaluation of the switching rates

We use the data sets shown in Fig. 3A to count the number of switching from one conductance level to another during a data set measurement (duration about 2 min) with the molecular chain exposed to light. Furthermore, we evaluate the rates of switching, which we calculate as follows: The switching rate f_{X-Y} from state X to state Y

$$f_{X-Y} = \frac{N_{X-Y}}{t_X}, \tag{1}$$

where N_{X-Y} is the total number of switching events from X to Y detected during the measurement and t_X is the total time spent by the molecular chain in state X during the measurement. Here, we distinguish the three states which presumably correspond to different number of defects on the chain: The high-conductive (HC) defect-free state, a low-conductive (LC) one-defect state and a non-conductive (NC) two-defect state. The numbers shown in Fig. 3E have been produced with the following threshold values of current chosen

to differentiate between the conductance states: 1.8×10^{-8} A marked the switch between no defect and one-defect state while 3.8×10^{-11} A marked the switch between one-defect and two-defect states. As we argue in the next subsection 1.3, no abrupt changes involving two defects, like switching from the HC to a NC state or back have been observed.

Each data set of current measured under constant conditions (constant chain lifting height, bias, intensity of light) consisted of 11,775 data points. The acquisition of one point took 10 ms. This sampling time of 10 ms limits our time resolution. The data shown in Fig 3E have been obtained at the bias of $V = 80$ mV.

In order to plot the switching rates as a function of bias, we needed an automated procedure to ascribe individual data points of $I(t)$ (representing the instantaneous current) to one of the distinct conductance levels. The constant threshold values as quoted above were suitable for the one particular value of bias only. The algorithm we used to process multiple data sets at varying bias has been as follows:

- We used KDE as described in the above subsection 1.1 to get the probability density of $\log(I)$ in each data set.
- We applied the `find_peaks` function of the `scipy.signal` Python library (see `https://docs.scipy.org/doc/scipy/reference/signal.html`) in order to identify peaks in the probability density; those peaks corresponded to the distinct conductance levels.
- A given data point $I(t)$ has been associated with a particular conductance state if the value $\log(I)$ has lied between `left_ips` and `right_ips` of the corresponding peak. The limiting values `left_ips` and `right_ips` were parameters returned by the `find_peaks` function called with `rel_height=1`.
- Dealing with ambiguity: If a particular value of I fulfilled the above condition for several probability-density peaks at once, the one that was closer to the measured current in the logarithmic scale was picked.
- Dealing with outliers: If there were any data points not attributed to any of the probability-density peaks and if these points were preceded by a data point attributed to state X and followed by a data point attributed to state Y, the event was still counted as a switch from X to Y.
- If there were any data points not attributed to any of the probability-density peaks and if these points were preceded by a data point attributed to state X and followed by a data point attributed to the same state X, these data points were still counted to the total time spent in state X.

1.3 Absence of the direct conductance switching between HC and NC states

Here, we argue that the conductance switching that involves a simultaneous annihilation or formation of two defects, i.e. switching from the high-conductive (HC) to the non-conductive (NC) state (0→2, HC→NC) or *vice versa* (2→0, NC→HC) most likely does not take place

in the experiments. The apparent 0→2 transitions we identified in our measured data can be explained as a result of two consecutive 0→1 and 1→2 transitions taking place in rapid succession, which we fail to distinguish because of finite time resolution in the experiments. Similarly, the apparent 2→0 transitions can be explained as a 2→1 transition quickly followed by a 1→0 transition. Given the sampling rate t_{int}=10 ms and denoting the observed rates of the 0→1, 1→2, 2→1, and 1→0 transitions as f_{0-1}, f_{1-2}, f_{2-1}, and f_{1-0}, respectively, the expected rates of fake HC→NC and NC→HC events will be

$$f_{0-2} = f_{0-1} f_{1-2} t_{\mathrm{int}}$$

and

$$f_{2-0} = f_{2-1} f_{1-0} t_{\mathrm{int}}.$$

Fig. S10 displays an example of the corresponding experimental data including the apparent rates of 0→2 (blue down-pointing triangles) and 2→0 (violet up-pointing triangles) switching rates. We compare them to the estimated rate of false detection (blue and violet asterisks). The agreement between the rates of empirically detected (triangles) and expected fake (asterisks) transitions suggests that in fact all apparent changes by two defects at once can be explain as false ones. Therefore, we conclude that the system can change by only one local defect being added or removed at a time. In Fig. S11, we plot the selected rates again, without the curves that corresponded to the double switching.

1.4 Conductance Model

We build our model from a chain of n one-electron sites, each characterized by a single energy level $-\epsilon_d$ (with respect to the Fermi level) and coupled to its nearest neighbors with a hopping parameter t (fig. S8a). The presence of a defect on a particular site is simulated by shifting the on site energy level by $-\Delta\epsilon_d$, that is, to $-(\epsilon_0 + \Delta\epsilon_d)$. We assume $\Delta\epsilon_d > 0$, so that the shift is downwards, away from the Fermi energy (fig. S8c). The requirement to reproduce the decay of electric current with chain length, $I \propto e^{-\lambda z}$ with $\lambda \approx 1.0$ nm^{-1}, gives $-\epsilon_d = -1.1$ eV for $t = 0.5$ eV. Of course, the experimental value of λ allows us to fix only one parameter of the model, ϵ_d or t, it does not uniquely determine the value of both simultaneously. Generally, any choice that conforms to $(\epsilon_d - 2t)/t \approx 0.2$ would be compatible with $\lambda \approx 1$ nm^{-1}. The expression $-(\epsilon_d - 2t)$ can be interpreted as the upper edge of the valence band (in the absence of defects and bias voltage). We made the particular choice of $t = 0.5$ eV and $\epsilon_d - 2t = 0.1$ eV because for $\epsilon_d - 2t < 0.1$ eV and $t < 0.5$ eV, strong non-linearity was observed in the simulated $I(V)$ dependence, especially at higher bias, $|V| > 0.1$ V, at odds with the measured $I(V)$ curves, while $t > 0.5$ eV would require extremely large $\Delta\epsilon_d$ to reproduce the conductance drop caused by the presence of a defect. $\Delta\epsilon_d \approx 12$ eV is needed to reproduce the separation between the conductance levels of defect-free (conductive) state and those of the single-defect (low-conductive) states, which was observed to be between two and three orders of magnitude.

Next, we introduce an experimentally observed asymmetry with respect to the polarity of the voltage applied across the molecular junction (fig. S8b,d). The asymmetry will be included in the model by defining nonequivalent coupling to the two electrodes, the surface and the tip, characterized by parameters Γ_{s} and Γ_{t}, respectively. These Γ_{s}, Γ_{t} define the

smearing of energies at the first and last site of the chain segment, caused by the coupling of these edge sites to the respective electrodes. Apart from this electron coupling to the electrodes, we need to specify how the electrostatic bias between the electrodes translates into the shift of the electron energy levels inside the chain. In the limit of very tight electrostatic coupling, the energy levels of the first and last sites in the chain will be pinned to the respective Fermi levels of the adjacent electrodes. So the first level shifts by $-V/2$ and the last level by $+V/2$ upon application of a total voltage V. The shift of energies on the other sites in between will be given by a linear ramp that interpolates between the shifts of the edges. If there is imperfect pinning to an electrode, the energy level of the neighboring site will shift by less than $|V/2|$. In principle, we could introduce two new parameters into the model so as to describe the electrostatic pinning, but we rather expect the pinning to be related to the electronic couplings Γ_s, Γ_t. We take the coupling of the molecular chain to the tip to be tighter as compared to its coupling to the substrate (which makes lifting of the chain by the tip possible), so $\Gamma_\mathrm{t} \geq \Gamma_\mathrm{s}$. Accordingly, we will assume a perfect pinning on the side of the tip, corresponding to the energy shift of

$$\Delta V_\mathrm{t} = +\frac{V}{2} \tag{2}$$

with applied bias, while we scale down the shift on the site adjacent to the substrate according to

$$\Delta V_\mathrm{s} = -\left(\frac{\Gamma_\mathrm{s}}{\Gamma_\mathrm{t} + \Gamma_\mathrm{s}}\right) V. \tag{3}$$

Taking all the above parameters into account, our model Hamiltonian will be

$$H_{ij} = \begin{cases} -\epsilon_d - \delta_i(\Delta\epsilon_d) + V_i & \text{for } i = j \\ t & \text{for } i \neq j \\ 0 & \text{otherwise,} \end{cases} \tag{4}$$

where $i, j = 1 \ldots n$ index the individual sites that comprise the simulated chain, $\delta_i = 0$ for an unperturbed site and $\delta_i = 1$ on a site with a defect. The bias-dependent ramp enters the Hamiltonian through the diagonal terms

$$V_i = \left[\Delta V_\mathrm{s}\,(n - i) + \Delta V_\mathrm{t}\,(i - 1)\right]/(n - 1). \tag{5}$$

We evaluate the current as a function of bias voltage in the following way. We find the retarded

$$\mathcal{G}_r(E) = (E - H - \Sigma_\mathrm{L} - \Sigma_\mathrm{R})^{-1} \tag{6}$$

and advanced

$$\mathcal{G}_a(E) = \mathcal{G}_r^\dagger(E) \tag{7}$$

Green's functions by matrix inversion, starting from the Hamiltonian Eq. (4). Here, the self-energy matrices are defined in terms of the coupling parameters Γ_L and Γ_R by setting

$$(\Sigma_\mathrm{L})_{11} = i\Gamma_\mathrm{L}, \tag{8}$$

and

$$(\Sigma_\mathrm{R})_{nn} = i\Gamma_\mathrm{R}, \tag{9}$$

with all other matrix elements being zero,

$$(\Sigma_{\rm L})_{ij} = 0 \quad (\text{for } i > 1 \text{ or } j > 1) \tag{10}$$

and

$$(\Sigma_{\rm R})_{ij} = 0 \quad (\text{for } i < n \text{ or } j < n). \tag{11}$$

$$I(V) = \int_{-eV/2}^{+eV/2} \mathrm{Tr}\left[\Sigma_{\rm L}\mathcal{G}_r(E)\Sigma_{\rm R}\mathcal{G}_a(E)\right] dE \tag{12}$$

The integral was performed with a numeric step of $dE = 1$ meV.

1.5 Interaction between low-spin defects

We asses the stability of various two-defect configurations by evaluating the DFT total energy of the Co-QDI chain containing two low-spin defects as a function of the distance between these two defects. The same hybrid functional was used in this calculation as for the other calculations in this work, namely the modified PBE0 with 40 % admixture of Fock exchange. The model was an infinite molecular chain, the structure of which repeated periodically after 8 molecular units. The 1D Brillouin zone corresponding to the supercell of 8 molecular units was sampled by 3 k-points. The supercell contained 2 low-spin Co atoms (as defect) and 6 high-spin C atoms as the unaltered environment between the defects. We have calculated the total energy for 4 cases which differ by the separation between the nearest defect sites: no high-spin Co, 1, 2, and 3 high-spin Co atoms between the defective low-spin Co atoms, respectively. The mutual orientation of the localized magnetic moments on Co atoms was maintained such that it corresponded to the lowest energy under the constrain given by the presence of the defects. In particular, the orientation of the two neighboring high or two neighboring low moments was opposite (antiferromagnetic) while a low-spin defect with a high-spin nearest neighbor had the same (ferromagnetic) orientation of their corresponding local moments. The energy of the last case with maximal separation between the defect sites

separation	$E_{\rm tot}$ (meV)
0	+2.823
1	+0.829
2	+0.120
3	0

Table S 1: Interaction energy of a pair of low-spin defects

was used as a reference with respect to which the other energies are compared. The results in Table S1 demonstrate a weak repulsion between the defects.

1.6 Exchange-functional dependence of magnetic states

The DFT calculation unfortunately cannot reliably predict the relative energies of different magnetic configurations of the molecules. As shown in Tab. S2, the difference of total energies per one molecular unit between the high-spin and the low-spin state depends on the portion

of Fock exchange in the PBE0 functional (α_X). Note that the standard choice in PBE0 is $\alpha_X = 0.25$. The high-spin state is correctly predicted to be the ground state with $\alpha_X \approx 0.4$ or higher.

PBE0 α_X	$E_\mathrm{HS} - E_\mathrm{LS}$ (meV)
0.25	+324.8
0.30	+119.2
0.35	+85.8
0.40	-23.0
0.50	-221.9

Table S 2: Dependence of the calculated relative energy of the two magnetic states on the exchange parameter of the hybrid exchange-correlation functional.

1.7 Alternative switching scenarios

An alternative explanation of the chain conductance switching could be light-driven conformational changes, such as the rotation of ligand units around the chain's longitudinal axis leading to alterations in Co atom coordination, or substantial modifications of the Co-N bonds. In scenarios involving such significant conformational shifts, one would expect these changes to significantly influence the shape of frequency shift traces recorded during the chain lifting. Fig. S12 displays frequency shift and the current signal acquired simultaneously while lifting a Co-QDI chain with an external light on ($\lambda = 600\,\mathrm{nm}$). While the current signal shows characteristic random switches between different conduction states during the course of the lift, the frequency shift signal remains stable, lacking any stochastic fluctuations that would indicate the presence of considerable conformational changes.

This point is further underscored by comparing the frequency shift traces obtained during chain pulling with the light turned on (indicating conductance switching) versus with the light off (where no conductance switching occurs), as depicted in Fig. S13. In both scenarios, we observe a consistent pattern of periodically occurring spikes, which represent the detachment of individual units from the surface. It's worth noting that while the periodicity of these spikes and the morphology of the intervening plateaus might vary slightly across different data sets due to minor variations in the junction's configuration, there are no substantial differences in their overall profiles. These observations strongly suggest that conductance switching is not the result of significant conformational alterations within the chain.

2 Supporting Figures

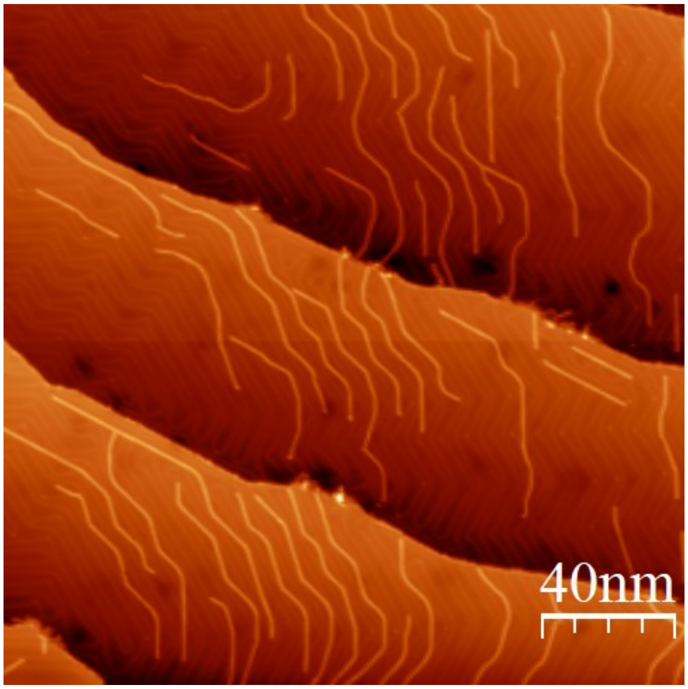

Fig. S 1: 200x200 nm STM image of the CoQDI chains on Au(111) at 100 mV, 10 pA setpoint.

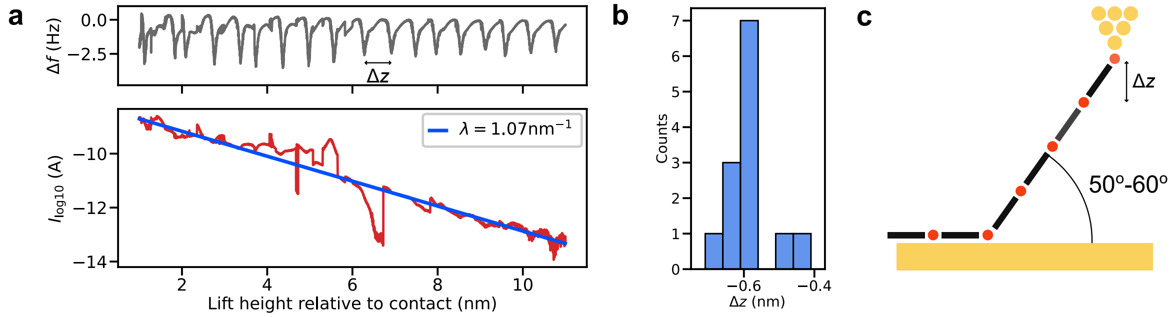

Fig. S 2: a) Upper: Characteristic Δf trace of a initial chain lift 12 nm from the contact height. The periodic spikes in the trace represent lifting of subsequent chain units (17-18 units lifted). Lower: Simultaneously acquired current signal trace exhibiting unusually small decay constant $\lambda = 1.7\,\text{nm}^{-1}$ for $I(h) = A * \exp(-\lambda * h)$ $(V = 1\,\text{mV})$. b) Histogram of the Δz between subsequent spikes in the Δf trace presented in a). We attribute the discrepancy between the median of the Δz (0.59 nm) and the calculated lattice vector of the CoQDI chain (~ 0.79 nm) to the fact that the chain forms during the initial lift an angle of $50° - 60°$ with the substrate as shown in c).

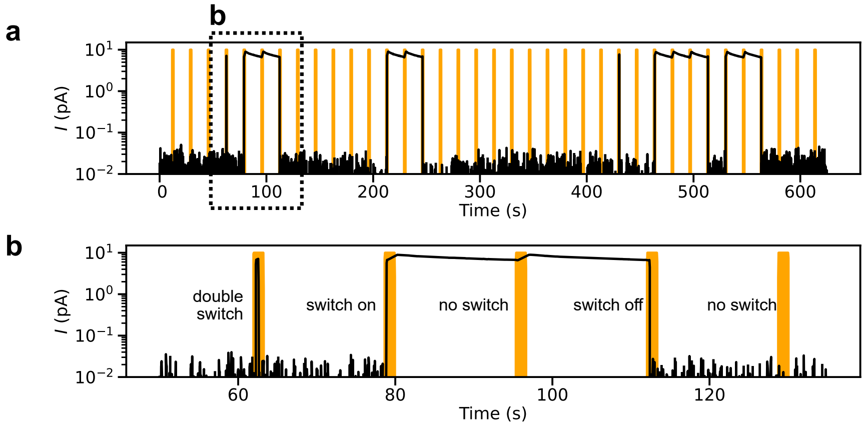

Fig. S 3: The experiment consists of cycles involving brief illumination (1.2 s) of the suspended CoQDI chain using a commercially available white diode (400 nm − 750 nm), followed by a waiting time of 15.5 s with the light switched off.a) The current signal trace, depicted in black, is recorded for approximately 10 min with the illumination pulses highlighted in yellow. $(V = 10\,\text{mV})$. b) A zoomed-in version of the trace from part a) shows that conductance switching occurs during the illumination period, while no switching is observed during the dark time. Once the light is switched off, the chain maintains the same conductance state until the next illumination interval (15.5 s). Then, the conductance is either switched again or remains unchanged. We rarely observe double switches within one illumination period.

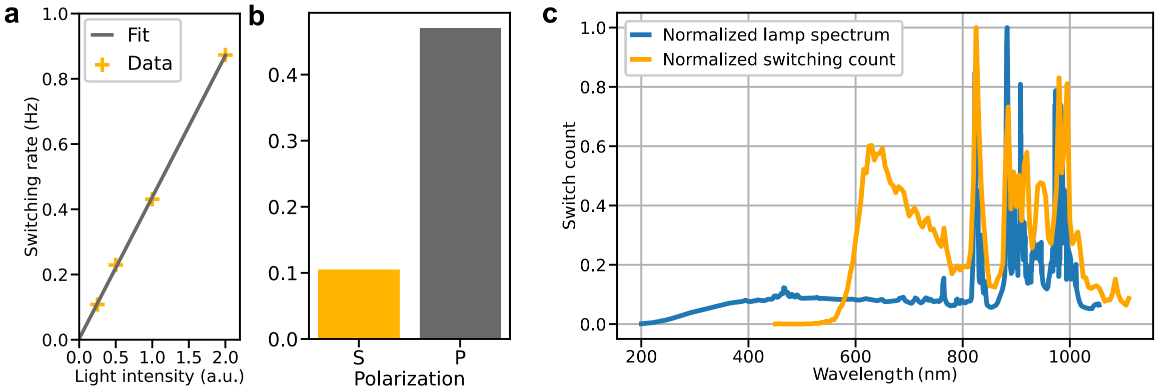

Fig. S 4: a) The switching rate increases linearly with the light intensity. The intensity is regulated by opening an exit slit on the Xe lamp monochromator at constant wavelength (600 nm). For each slit opening, the switching rate is estimated from the current signal recorded for 16.5 min. b) Switching rate obtained on the same chain at the same lift height for p and s-polarized light (600 nm). c) The normalized switching count shows the dependence of conductance switching on the wavelength of the incoming light. The switch counts were extracted from current signal traces (3 min) for subsequent 5 nm increments in the light wavelength. The threshold around 550 nm correlates with the reflectance edge of gold. For comparison, we plot the normalized lamp spectrum (*39*) to explain the origin of the peaks at the wavelengths above 800 nmA.

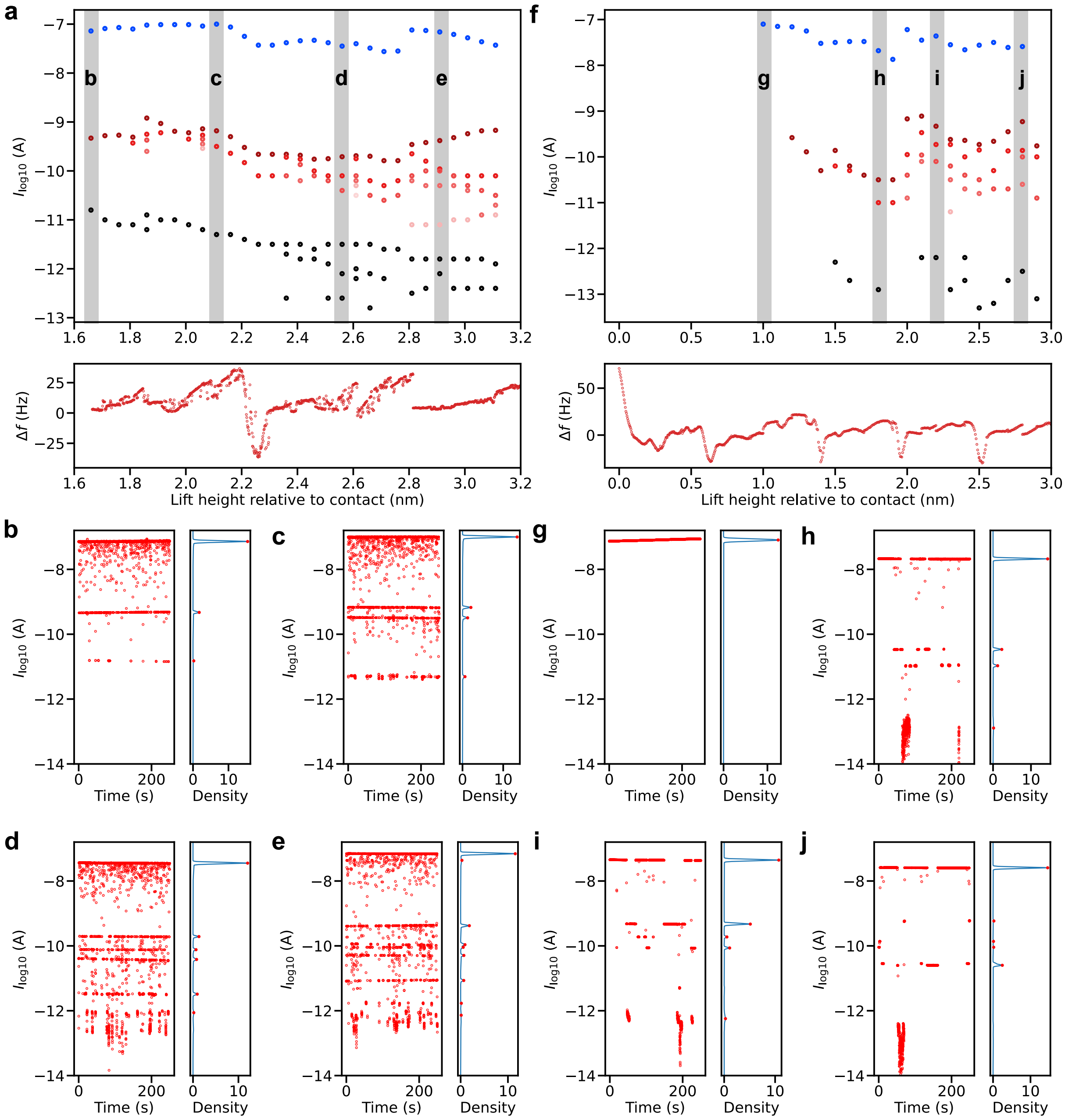

Fig. S 5: a) and f) Independent (different tip, different chain) experiments showing evolution of conductance levels with the lift height, extracted from the Kernel density estimate (KDE) for each lift increment and respective Δf trace recorded during the lifting. b), c), d), e) and g), h), i), j), respectively, are exemplary current vs time traces (marked in a) and f)) along with their KDE analysis. After reaching a critical length, we observe three different sets of conductive states: HC, LC and NC states. While there is only one HC state independent of the length of the suspended chain, the number of LC states increases linearly with the number of units of the suspended chain. (V = 150 mV and $t_{\text{int}} = 50\,\text{ms}$, resp. V = 20 mV and $t_{\text{int}} = 50\,\text{ms}$, Xe lamp $\lambda = 600\,\text{nm}$).

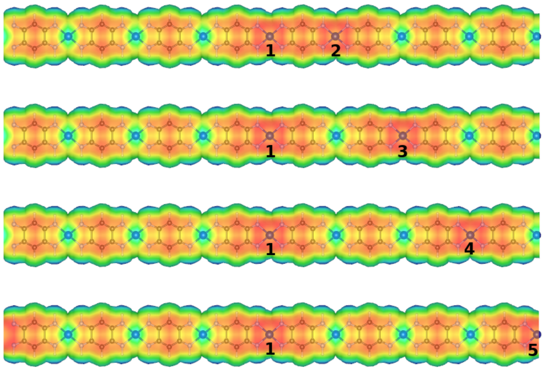

Fig. S 6: Molecular electrostatic potential surface (MEPS, that is, color-coded electrostatic potential on a surface of constant electron density, here 0.01 eÅ^{-3}) of chains with two defects in various configurations. The separation between the two defect increases from top to bottom. The position of the first defect is denoted as site 1, the sites of the second defect are 2, 3, 4, and 5 for separations of no high-spin Co, 1, 2, and 3 high-spin Co sites between the low-spin defect sites, respectively. The defective sites can be identified by a partial negative charge visualized as a more negative electrostatic potential (red).

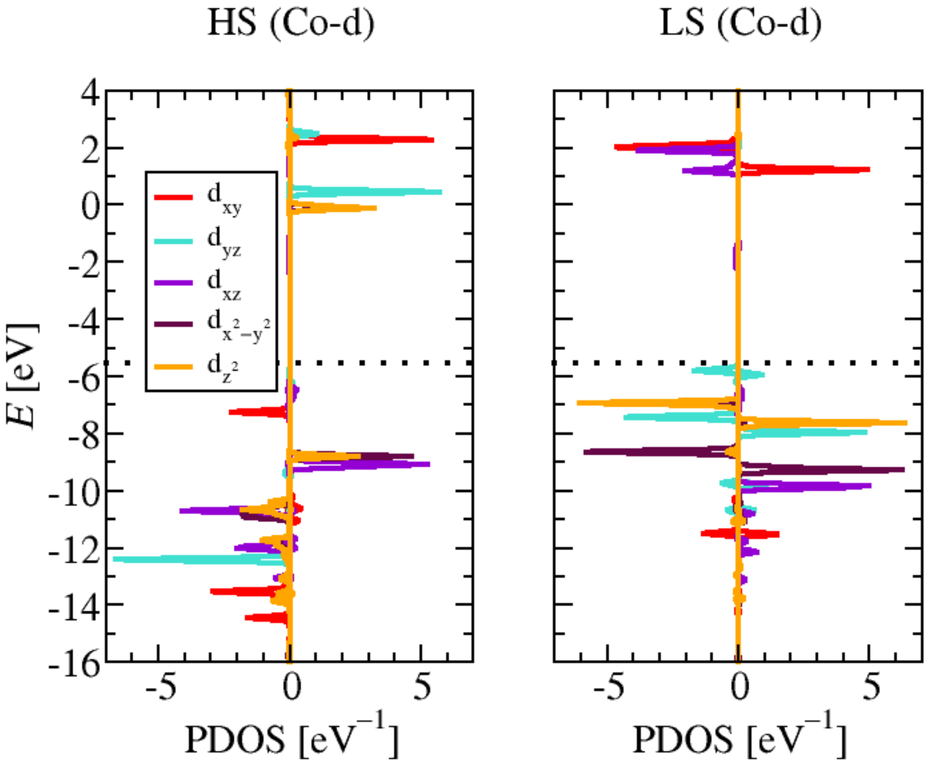

Fig. S 7: Density of states (PDOS) projected on individual d orbitals of a Co atom in the Co-QDI chain. The plot has been calculated for an infinite chain with 8-unit periodicity, consisting of 7 molecular unit long rows of high-spin Co atoms with the antiferromagnetic arrangement of their magnetic moments, interrupted by 1 low-spin Co defect (with the magnetic moment ferromagnetically aligned to the two neighboring low-spin Co atoms). Left: PDOS on the high-spin Co atom most distant from the low-spin defect. Right: PDOS on the low-spin Co. The calculation was done using a modified PBE0 functional with 40 % admixture of Fock exchange and 3 k-points sampling the 1D Brillouin zone corresponding to the 8-molecular-units-long supercell.

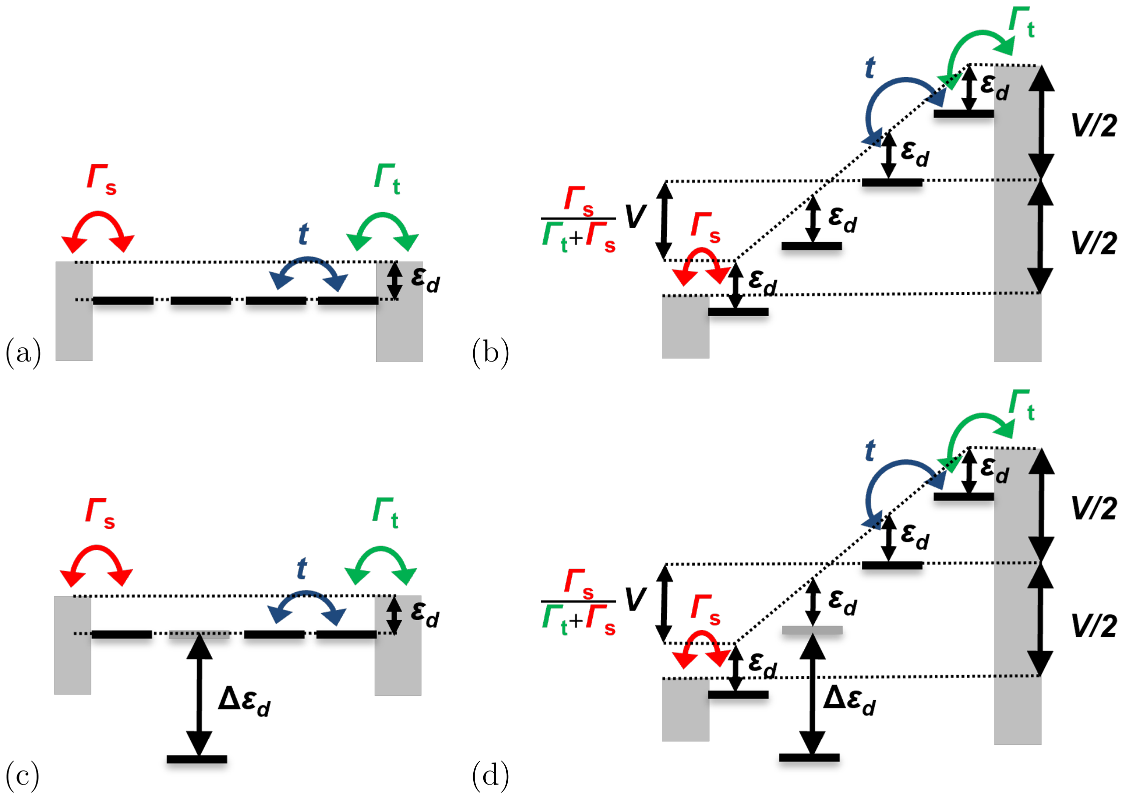

Fig. S 8: Graphic sketch of the transport model (a) without bias voltage applied and without local defects, (b) with bias voltage V manifest as a potential-energy ramp, still without local defects, (c) with (light-induced) local defects, no bias, (d) a general configuration with defects and a bias voltage V. The case shown is for $n = 4$ sites and one defect (on the 2nd site). Parameters of the model: the unperturbed on-site energy ϵ_d, the change of on-site energy caused by the defect $\Delta\epsilon_d$, inter-site coupling (hopping) t, coupling of edge sites with the electrodes Γ_s and Γ_t.

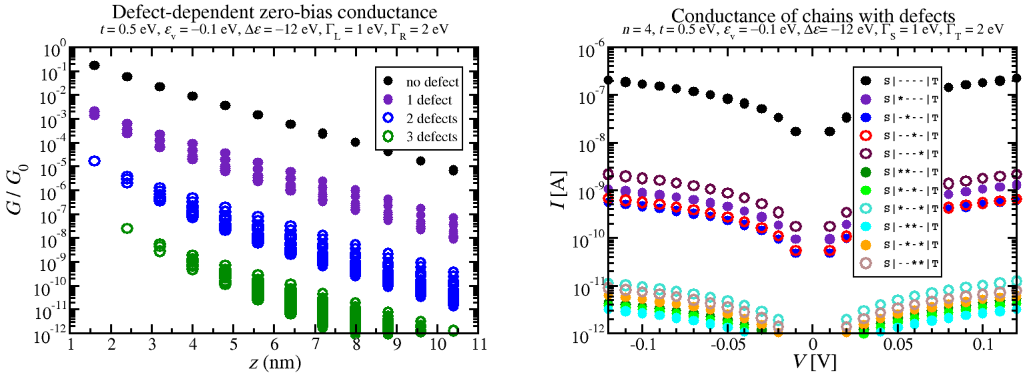

Fig. S 9: Left: Simulated $G(z)$ curves (conductance decay with chain length) for a chain with no defect and chains with one and two defects at various positions. Same color used for states with the same number of defects, independent of the placement of the defective site(s). Right: Simulated $I(V)$ curves (current as a function of bias) for a chain $n = 4$ units long. Different symbols correspond to states which differ by the number and position of defects. The simulations have been done with the following choice of parameters: $t = 0.5$ eV, $E_0 = 1.1$ eV, $\Delta E_d = 12$ eV, $\Gamma_\mathrm{s} = 1.00$ eV, $\Gamma_\mathrm{t} = 2.00$ eV.

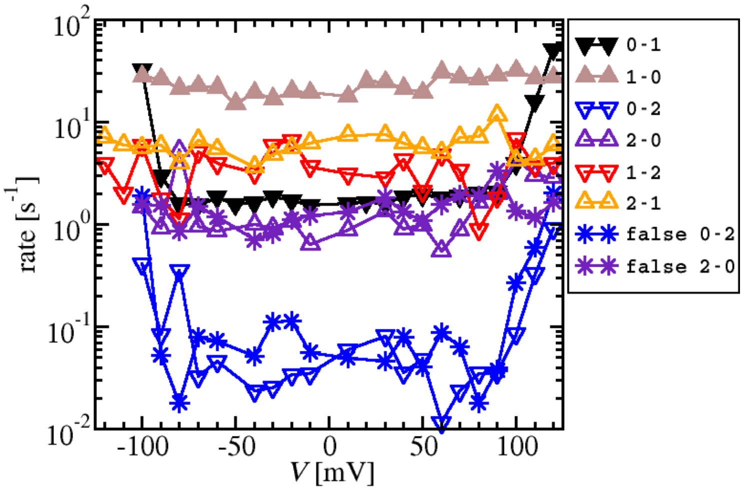

Fig. S 10: Analysis of apparent switching from HC to NC and back, which involves a change by two defects. Bias dependence of the switching rates with 4 units lift. Triangles: Empirical switching rate taken directly from experiment as the number of detected transitions divided by measuring time. Asterisks: Predicted level of false 0→2 (HS→NS) and 2→0 (NS→HS) transitions from consecutive one-defect transitions. For details see discussion in Chapter 1.3.

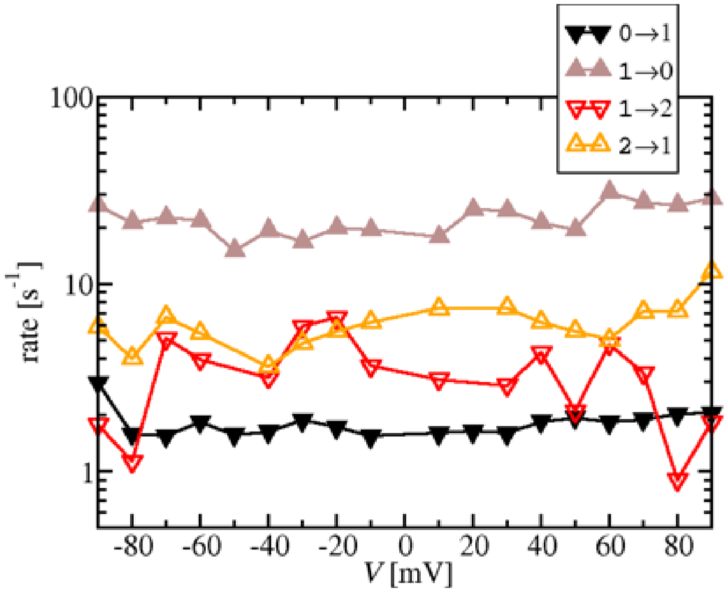

Fig. S 11: Bias dependence of the switching rates with 4 unit lift. The characteristic sets of states are HC=0, LC=1 and NC=2.

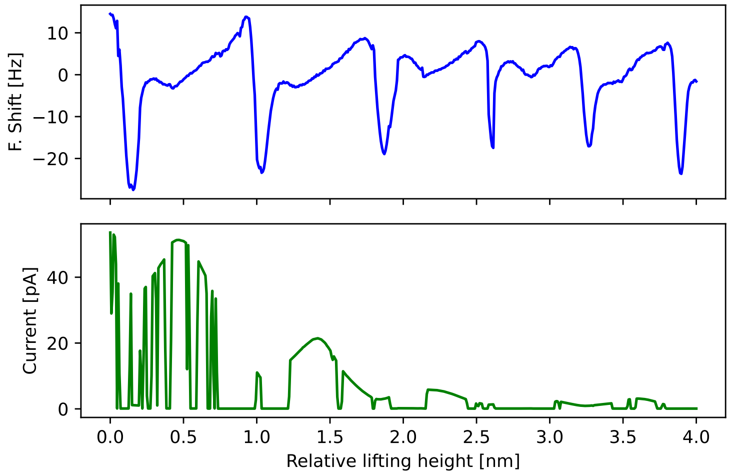

Fig. S 12: Simultaneously recorded frequency shift (upper panel) and current (lower panel) signals during controlled lifting of chain with the light on ($\lambda = 600\,\mathrm{nm}$, $V = 10\,\mathrm{mV}$).

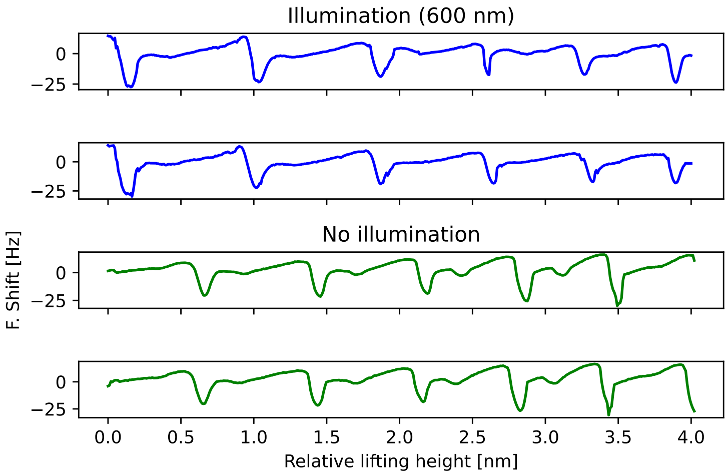

Fig. S 13: Comparison of several frequency shift traces during the lifting of different chains with light on (two upper panels in blue) and without external light (two lower panels in green).
